# Pontocerebellar Hypoplasia Type 3 With Two Novel *PCLO* Gene Mutations: A Case Report

**DOI:** 10.1155/crpe/1955363

**Published:** 2025-07-07

**Authors:** Sethapong Lertsakulbunlue, Panithi Piyachon, Pitchaya Pichantianchai, Thanapat Chivaruangrot, Piradee Suwanpakdee, Boonchai Boonyawat

**Affiliations:** ^1^Department of Pharmacology, Phramongkutklao College of Medicine, Bangkok, Thailand; ^2^Medical Cadet, Phramongkutklao College of Medicine, Bangkok, Thailand; ^3^Department of Pediatrics, Phramongkutklao College of Medicine, Bangkok, Thailand

**Keywords:** *PCLO*, Piccolo, pontocerebellar hypoplasia, Thailand

## Abstract

Pontocerebellar hypoplasia Type III (PCH3) is a rare, autosomal recessive neurodegenerative disorder linked to mutations in the *PCLO* gene, previously reported only in Omani populations. It presents with progressive microcephaly, intractable epilepsy, optic atrophy, and severe developmental delay. Here, we report the first documented case of PCH3 in an 8-year-old Thai girl with two novel *PCLO* truncation mutations. The patient presented with intractable epilepsy from 2 months of age and severe global developmental delay. Whole exome sequencing identified compound heterozygous mutations in the *PCLO* gene: c.9018_9037del (p.Tyr3007Ter) and c.8456del (p.Ala2819GlufsTer2), both of which were inherited from heterozygous parents. These mutations are predicted to result in a loss of Piccolo protein function. This case expands the mutational spectrum of *PCLO*-related PCH3 and highlights the importance of advanced molecular diagnostics in understanding and managing this rare neurodegenerative disorder. Given the lack of curative therapies, early genetic diagnosis is crucial in guiding patient care and genetic counseling.

## 1. Introduction

Pontocerebellar hypoplasia (PCH) is a diverse group of rare genetic neurodegenerative disorders primarily affecting the pons and cerebellum [1]. In 2024, 17 types of PCH associated with 25 genes have been identified [2]. PCH Type III (PCH3), also recognized as cerebellar atrophy with progressive microcephaly (CLAM), is a rare form characterized by neonatal hypotonia, progressive microcephaly, seizures starting in the first year of life, optic atrophy, and severe neurodevelopmental delay [3]. However, these clinical manifestations are nonspecific. PCH3 was first described in the Omani family in 2003 and was subsequently identified as caused by a loss-of-function mutation in the Piccolo (*PCLO*) gene, located at 7q21.11 [4–6].

Herein, we report the first case of an 8-year-old Thai girl presenting with intractable epilepsy starting at the age of 2 months and severe global developmental delay. Compound heterozygous truncating mutations in the *PCLO* gene were identified and inherited from both heterozygous parents, causing the diagnosis of PCH3 in our patient.

## 2. Case Report

An 8-year-old Thai girl presented to Phramongkutklao Hospital with intractable epilepsy, which began at 2 months of age. She was born prematurely at 35 weeks of gestation via cesarean section due to fetal distress and premature rupture of membranes. She had a neonatal course complicated by moderate birth asphyxia and sepsis. Her birth weight was 2840 g. She is the child of nonconsanguineous parents. Since birth, she exhibited generalized hypotonia and subsequently developed severe global developmental delay.

At 8 months of age, physical examination revealed an occipitofrontal circumference (OFC) of 38 cm (< 3rd percentile), weight of 7775 g (50th–75th percentile), and length of 68 cm (50th–75th percentile). Craniofacial features included marked microcephaly, prominent eyes, and low-set ears. Neurological examination demonstrated poor responsiveness, truncal hypotonia, increased deep tendon reflexes, and spasticity with contractures in all extremities. Fundoscopic examination showed pale optic atrophy.

**Figure 1:**
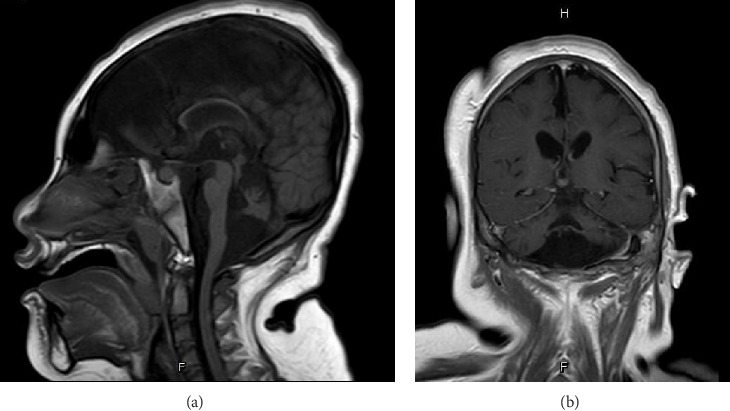
Brain MRI of the patient at 1 year of age. (a) Midsagittal T1-weighted image shows a thin corpus callosum, small pons, thinning of the medulla oblongata, and a hypoplastic cerebellar vermis. (b) Coronal T1-weighted image demonstrates a small cerebellar vermis and hypoplastic cerebellar hemispheres.

Basic laboratory evaluations, including hematological profiles and serum biochemical parameters, were within normal limits. Inborn error of metabolism was initially excluded by normal levels of serum ammonia, serum lactate, plasma amino acids and urine organic acids. Chromosomal analysis demonstrated a normal female karyotype (46, XX). Brain MRI performed at 1 year of age revealed a small brainstem, hypoplastic vermis, bilateral hippocampal atrophy, and reduced cerebellar hemispheres with widened choroid fissures (Figures 1(a) and 1(b)). The corpus callosum appeared thin, and diffuse cerebral atrophy was noted.

Regarding her epilepsy, generalized tonic–clonic seizures occurring 3-4 times per day were first observed at the age of 2 months. Electroencephalography (EEG) revealed frequent sharp waves originating from the bilateral parieto-occipital regions. Phenobarbital was initiated as the first antiepileptic drug, but a generalized maculopapular rash developed within a few days of treatment. Consequently, phenobarbital was discontinued and replaced with sodium valproate. Despite treatment, the seizures remained difficult to control. Topiramate and levetiracetam were added at 6 months of age. Sodium valproate was later replaced with perampanel, and carbamazepine was introduced at 2 and 5 years of age, respectively. Seizure control was eventually achieved with a combination of four antiepileptic drugs: topiramate, levetiracetam, perampanel, and carbamazepine. However, she has remained bedridden since infancy.

## 3. Molecular Methods and Results

After obtaining informed consent, blood samples were collected from the patient and the parents. Genomic DNA was extracted from peripheral blood using the QIAmp DNA Blood Mini Kit (QIAGEN, Germany). Whole exome sequencing was conducted using the SureSelect Human All Exon V7 (Agilent) for library preparation, with sequencing performed on the NovaSeq 6000 (Illumina) (Macrogen, Korea). The sequences were aligned to the human genome reference sequence (GRCh37) using BWA (Burrows-Wheeler Aligner) and GATK (Genome Analysis Toolkit), respectively.

**Figure 2:**
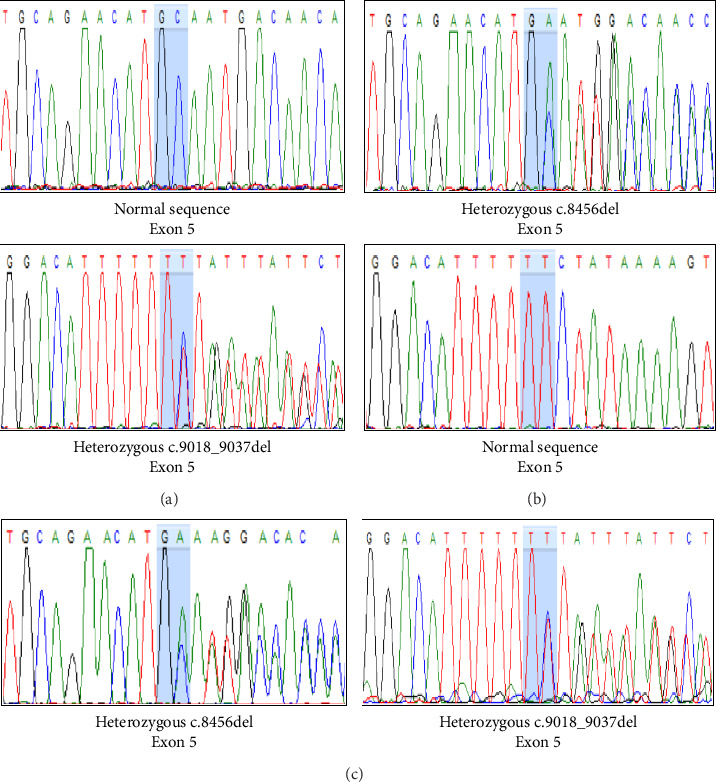
Sanger sequencing of Exon 5 of the *PCLO* gene in the patient, father, and mother. A heterozygous c.9018_9037del mutation was identified in the father (a), a heterozygous c.8456del mutation was identified in the mother (b), and a compound heterozygous mutation consisting of both c.8456del and c.9018_9037del was identified in the patient (c).

Variant analysis identified a compound heterozygous of two novel truncation variants in the *PCLO* gene. The first variant, c.9018_9037del (p.Tyr3007Ter), involved a 21-bp deletion from nucleotide position 9018 to 9037 in Exon 5, leading to a premature termination codon at the amino acid position 3007. The second variant, a frameshift c.8456del (p.Ala2819GlufsTer2) mutation in Exon 5, resulted in a premature stop codon at the amino acid position 2821. Sanger sequencing confirmed our patient's and her parents' autosomal recessive inheritance pattern (Figure 2). Neither variant was identified in the 1000G, ExAC, and gnomAD population databases. According to the 2015 ACMG classification [7], both variants were classified as “pathogenic” due to PVS1, PM2, and PM3 criteria.

## 4. Discussion

PCH is a genetically heterogeneous neurodevelopmental disorder and was first classified into two subtypes in 1993. The classification was based on the presence (PCH1) or absence (PCH2) of motor neuron degeneration in the anterior horn of the spinal cord [8]. To date, 17 subtypes of PCH associated with 25 genes have been identified. PCH3 is an extremely rare subtype characterized by nonspecific clinical features and MRI findings of a small cerebellum and pons, which can lead to potential underdiagnosis of this condition [5].

The PCH3 phenotype spectrums encompass progressive microcephaly, seizures starting within the first year of life, nonspecific facial dysmorphisms, optic atrophy, and severe developmental delay [2]. Our patient's early-onset, refractory seizures at 2 months and global developmental delay align with previous reports [6]. Brain MRI findings in PCH3 typically show severe cerebellar hypoplasia, particularly in the vermis, significant pontine hypoplasia with flattened ventral pons, and a thin corpus callosum. Compared to PCH1 and PCH2, cerebellar and pontine hypoplasia in PCH3 is less extreme [9]. In addition to the classical clinical features of PCH3, all the characteristic brain MRI findings in PCH3 were also identified in our patient (Figure 1).

PCH3 was first reported in the Omani families in 2003, suggesting autosomal recessive inheritance.  Table 1 compares the characteristics of the previously reported patients from Omani families with those of the present case in this study. Evidence from two consanguineous families maps the genetic loci of PCH3 to chromosome 7q11-21, which was finally identified as the *PCLO* gene [4, 5]. The *PCLO* gene encodes Piccolo, a presynaptic scaffolding protein essential for synaptic vesicle trafficking and neurotransmitter release [10]. It regulates actin-associated vesicle movement and coordinates exocytosis and endocytosis at the presynaptic active zone, highlighting its critical role in neural communication and synaptic integrity [2, 10]. *PCLO* is highly expressed in the developing human brain, and its loss leads to synaptic dysfunction and widespread neuronal degeneration in both the cerebrum and cerebellum. As synaptic activity is essential for preventing apoptosis [11], *PCLO* mutations may trigger apoptotic pathways, contributing to neuronal loss [5, 12]. Supporting this, Piccolo knockout mice exhibit cerebellar network dysfunction and phenotypes resembling PCH3 [6].

In this study, we identified a compound heterozygous of two novel frameshift mutations in Exon 5 of the *PCLO* gene: c.9018_9037del (p.Tyr3007Ter) and c.8456del (p.Ala2819GlufsTer2). Both mutations introduce premature termination codons and are predicted to result in nonsense-mediated mRNA decay, leading to complete loss of Piccolo protein. Until now, only a c.10624C > T (p.Arg3542Ter) mutation in Exon 6 of the *PCLO* gene has been reported in PCH3, found in Omani patients [5]. This mutation is predicted to disrupt the PDZ and C2 domains at the C-terminus of the Piccolo protein. Our patient's mutations are located proximally and are likewise predicted to impair these functional domains. These findings expand the mutational spectrum of the *PCLO* gene and further support its critical role in the pathogenesis of PCH3.

### 4.1. Genetic Counseling and Family Planning

At present, PCH remains incurable. Therapeutic interventions are confined to palliative measures. The lifespan of individuals diagnosed with PCH varies, extending from the neonatal phase to the late twenties, although a significant proportion of these patients die in childhood [2]. Given the autosomal recessive inheritance pattern of PCH3, there is a 25% recurrence risk in each pregnancy for couples in which both partners are carriers. Therefore, comprehensive genetic counselling is essential. For families in which the pathogenic variant has been identified, reproductive options such as preimplantation genetic diagnosis or prenatal testing should be offered. These approaches are particularly important given the high recurrence risk and severity of the disorder. It should be noted that prenatal detection of PCH via ultrasound is often unreliable, as cerebellar abnormalities may not be apparent during routine second-trimester screening [13]. Thus, molecular diagnostic strategies remain the most reliable tools for early detection and informed reproductive decision-making.

## 5. Conclusion

A decade after the last homozygous nonsense *PCLO* mutation report among Omanis, we describe the first documented case of an infant presenting with a PCH3, confirmed by two novel *PCLO* truncation mutations in Asia. Although PCH remains incurable to date, advanced molecular genetic diagnosis could significantly benefit families affected by PCH3. Our case underscores the identification of two novel genomic variants in the *PCLO* gene and further reinforces the association of PCH3 with a mutation in the *PCLO* gene.

## Figures and Tables

**Table 1:** Comparison between the first 4 reported pontocerebellar hypoplasia Type III patients from Oman and the present patient in this study.

Characteristics	An Omani family with 4 affected individuals^∗^	Patient in this study^∗∗^
Perinatal information	• No perinatal complication	• Gestational age: 35 weeks • Moderate birth asphyxia and sepsis

Age of onset	• 8 months to 1-year-old	• 2-month-old

Age of first diagnosis	• 11-year-old (age of the first diagnosed patient)	• 8-year-old

Initial presentation	• Severe global developmental delay • Seizures	• Severe global developmental delay • Generalized hypotonia • Seizures

Physical examination	• Micro-/brachycephaly • Prominent eyes • Low-set ears • Uplift earlobes • Spindle-shaped fingers • Short stature • Poorly developed scrotum • Poor responsiveness • Truncal hypotonia • Increased deep tendon reflexes • Pale optic atrophy	• Microcephaly • Prominent eyes • Low-set ears • Poor responsiveness • Truncal hypotonia • Increased deep tendon reflex • Spasticity of the extremities • Pale optic atrophy

Family history	• Consanguineous	• Nonconsanguineous

Electroencephalogram (EEG)	• Sharp discharges from the temporal regions bilaterally	• Frequent sharp waves from bilateral parieto-occipital regions

Brain MRI	• Diffuse atrophy of the cerebrum, cerebellum, and brainstem • Diminished white matter volume • Thin corpus callosum • The state of myelination was age-appropriate	• Small brainstems, hypoplastic vermis, bilateral hippocampal atrophy, and reduced cerebellar hemispheres with widened choroid fissures • Diffuse cerebral atrophy • Thin corpus callosum

Molecular testing (*PCLO* gene mutation)	• Homozygous c.10624C > T (Arg3542Ter) mutation in Exon 6	• Compound heterozygous of c.9018_9037del (p.Tyr3007Ter) and c.8456del (p.Ala2819GlufsTer2) mutation in Exon 5

Current medication	• Not reported	• Levetiracetam • Topiramate • Carbamazepine • Perampanel

^∗^Information from Omani patients is gathered from the previous studies of Rajab et al. [4, 5].

^∗∗^Key differences between the patient in this study and those from the Omani family include earlier onset, earlier diagnosis, and compound heterozygous mutations in Exon 5, inherited from nonconsanguineous parents.

## Data Availability

The data from this study can be made available upon a reasonable request to the corresponding author.
